# On the Physics Required for Prediction of Long Term Performance of Polymers and Their Composites

**DOI:** 10.6028/jres.099.014

**Published:** 1994

**Authors:** Gregory B. McKenna

**Affiliations:** National Institute of Standards and Technology, Gaithersburg, MD 20899-0001

**Keywords:** composites, constitutive equations, glasses, material clocks, nonlinear viscoelasticity performance, solid polymers

## Abstract

The long term performance of polymers and their composites is an important aspect of their increasing use in engineering applications. Temporal, thermal, and mechanical stresses can all contribute to the deterioration of performance. Here we examine the concepts of the physics of glassy polymers and how they are important in developing constitutive equations that describe their volume/temperature/stress time response. The understanding of such response forms the basis of the prediction of long term performance.

## 1. Introduction

When a glass forming liquid, such as a polymer melt or rubber, is cooled there is some temperature at which the molecular mobility decreases enough that the material’s thermodynamic state or structure cannot attain equilibrium in the time scale of the experiment (determined by the cooling rate) [1–3]. Below this temperature, generally referred to as the glass transition temperature *T*_g_, glassy materials are unstable with the result that their properties continuously evolve towards a temporally distant equilibrium. Efficient design with and use of polymeric materials requires an understanding of the physics underlying the structural recovery and the impact of the changing thermodynamic state on the mechanical properties of the material. In what follows we attempt to put into perspective the interrelationships between the physics of glasses, how it influences material performance and how we model material response using nonlinear constitutive equations. Additionally, we will describe some of the implications of structural recovery in polymers for composite materials performance. Finally, we will discuss the importance of developing methods to combine computer codes with nonlinear constitutive law modeling to material processing and performance prediction.

## 2. Phenomenology of Glasses

**Fig. 1:**
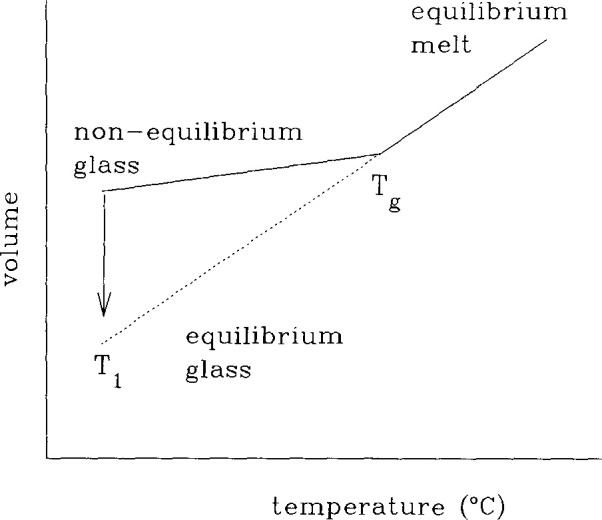
Schematic representation of the volume-temperature behavior of a glass forming material.

Referring to Fig. 1, the *glass transition event* can be defined in terms of a volume-temperature surface in a cooling experiment. At a given cooling rate, *q* = −d*T*/d*t*, the volume begins to depart from the equilibrium volume at a characteristic temperature *T = T*_g_ referred to as the *glass transition temperature*. If the magnitude of the cooling rate decreases, *T*_g_ decreases {*T*_g_(*q*_2_) < *T*_g_(*q*_1_) for *q*_2_ < *q*_1_}.

**Fig. 2:**
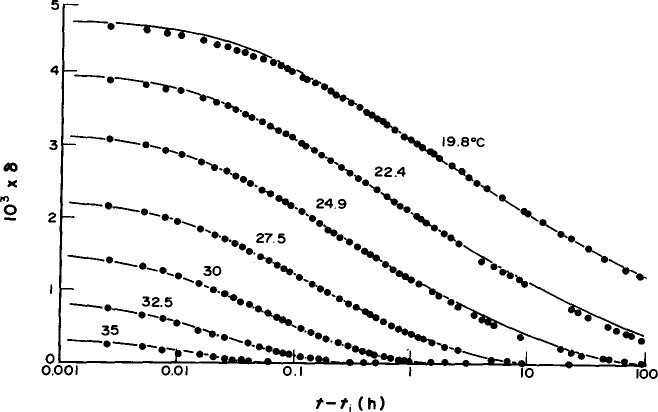
Isothermal contraction of glucose glass after quenching from *T*_0_ = 40 °C to different temperatures, as indicated (after Ref. [1], with permission).

If one cools a glass as depicted in Fig. 1 and then keeps the temperature fixed the volume of the material evolves spontaneously towards equilibrium, as represented by the arrow at *T*_1_. When the temperature is decreased rapidly in a *T-jump* experiment the *volume recovery* response looks like that depicted in Fig. 2 where the *volume departure from equilibrium δ*_v_ = (*ν*−*ν*_∞_)/*ν*_∞_ is plotted against the logarithm of the time after the temperature change. Note that *ν* is the (specific) volume at time *t* and *ν*_∞_ is the equilibrium volume. Similar results are obtained if one measures enthalpy instead of volume [4]. In general, one can refer to the evolution of the glassy polymer’s thermodynamic state towards equilibrium as *structural recovery* [4–6].

**Fig. 3:**
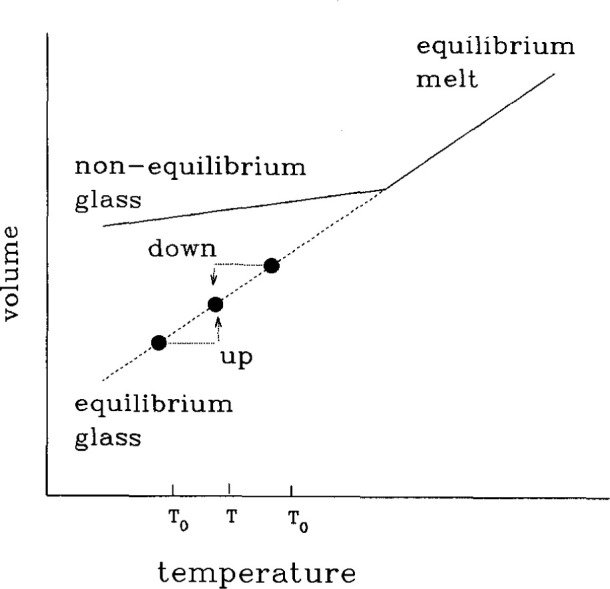
Volume-temperature schematic of the asymmetry of approach experiment. (See text for discussion.)

**Fig. 4:**
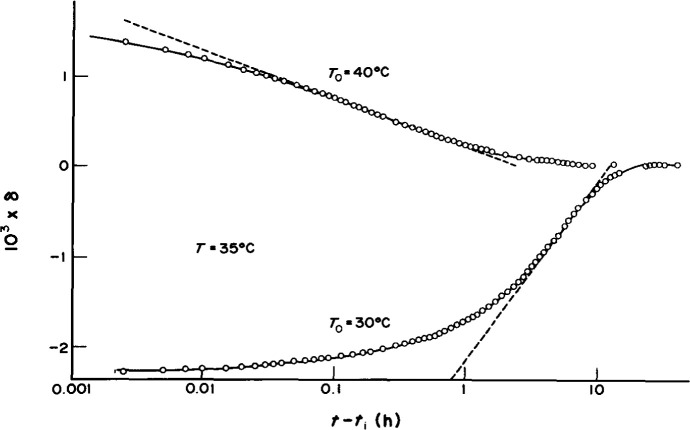
Expansion and contraction isotherms for poly(vinyl acetate) glass after heating and cooling from *T*_0_ = *T* ± 5 °C. This plot shows the asymmetry of the expansion and contraction isotherms (after Ref. [1], with permission).

*Asymmetry of approach* [1,3–6] experiments demonstrate the inherent nonlinearity of the structural recovery process. In these experiments, referring to Fig. 3, one equilibrates the glass prior to performing T-jump experiments by either increasing or decreasing the temperature. When one jumps from *T*_0_ *= T* − Δ*T* (up-jump) or *To = T +* Δ*T* (down-jump) to *T*, one finds that the response from below *T* is significantly different from that when the jump is from above *T*. This is readily seen in Fig. 4 in which up- and down-jump histories are depicted for a polymer glass. The asymmetry of approach to equilibrium is obvious. The nonlinear behavior was observed early by Tool [6] in working with inorganic glasses and has been attributed to a dependence of mobility on the current structure of the glass. Thus, in down-jump experiments, where the rate of volume recovery decreases with increasing time after the quench, the response is *autoretarded*, while in the up-jump the rate of volume recovery increases with increasing time and the response is *autocatalytic* [1]. As described subsequently, the dependence of the molecular mobility on the current glassy structure is one of the “essential ingredients” required to model the phenomenology of glassy behavior [3].

**Fig. 5:**
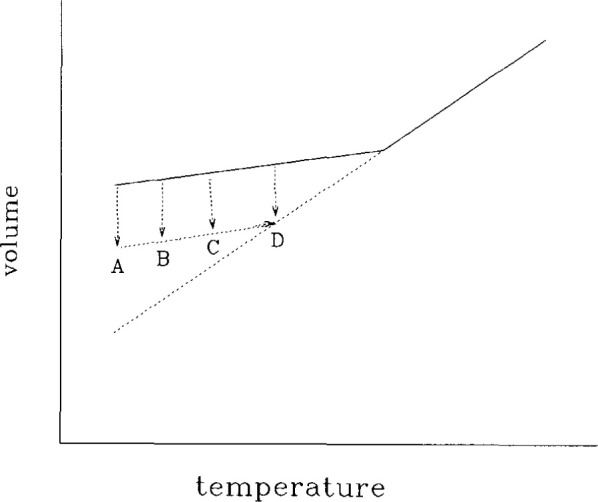
Schematic of volume-temperature history used in memory experiment (see text for discussion).

**Fig. 6:**
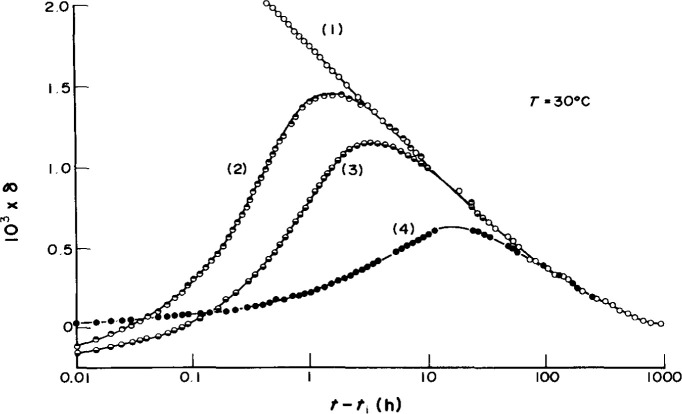
Isothermal evolution at *T*_0_ = 30 °C for poly(vinyl acetate) showing memory effect: (1) quench from 40 °C to 30 °C; (2) quench from 40 °C to 10 °C for 160 h followed by up-jump to 30 °C; (3) quench from 40 °C to 15 °C for 140 h followed by up-jump to 30 °C; (4) quench from 40 °C to 25 °C for 90 h followed by up-jump to 30 °C. Note that at short times *δ* ≈ 0. (After Ref. 1, with permission.)

The so-called *memory effect* is a manifestation of another essential ingredient required to describe glassy behavior. In such experiments one applies multiple temperature jumps to the sample. If we refer to Figs. 5 and 6 the most dramatic effect is seen when the experiment is performed such that the sample is first subjected to a down-jump to *T*_1_ (A, B, C in Fig. 5) and allowed to “age” isothermally until the volume extrapolated along the glassy thermal expansion line is the same as the equilibrium volume at *T*_0_ (D in Fig. 5). The sample is then subjected to an up-jump to *T*_0_ and the resulting initial departure from equilibrium *δ*_v_ is near to zero. As seen in Fig. 6, the glass does not remain in equilibrium, rather, it “remembers” its prior history and *δ*_v_ goes through a maximum before it approaches the response of a down-jump directly to *T*_0_ at long times. Such behavior cannot be explained without invoking a *nonexponential retardation function* to describe the behavior. The nonexponential function can either be represented as a *sum of exponentials* (distribution of retardation times) or using a Kolrausch [7]-Williams-Watts [8] (KWW) function (stretched exponential). The usefulness of these functions is described subsequently.

**Fig. 7:**
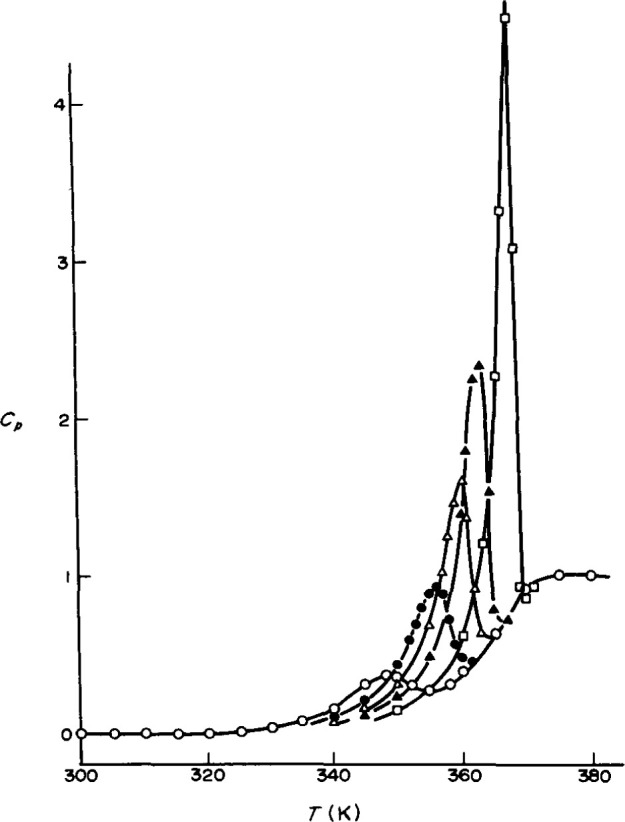
DSC curves calculated using a Narayanaswamy [9]-Moynihan [10]-type phenomenological model for glasses subjected to difference annealing times *t*_e_ at 60 °C. (○) 1 h; (●) 1 day; (Δ) 1 week; (■) 1 month; (□) 1 year. This demonstrates how complex features in DSC traces can occur due to the nonlinearity in the structural recovery response (after Ref. [46], with permission).

In the final paragraph of this section we discuss briefly the problems of performing *nonisothermal* types of experiments. The experiments described above, while using two or more temperatures were *isothermal* in the sense that the response of interest was observed at constant temperature. In those experiments we observed both the material nonlinearity (asymmetry of approach) and a manifestation of the nonexponentiality of the fundamental retardation response (memory effect). In nonisothermal experiments, one might expect that manifestations of these effects could lead to apparently interesting behaviors that truly were simple evidences of the nonlinearity and nonexponentiality of behaviors. Thus, in a differential scanning calorimetry experiment, for example, one follows the heat capacity *C_p_* as temperature is changed. Often one observes peaks, as shown in Fig. 7. The question then arises as to how to interpret such peaks and the possible effects of the nonlinear behavior of glasses need to be included in any interpretation of the results. Similar comments hold for other experimental methods such as thermomechanical analysis (TMA) or dynamical mechanical analysis (DMA) in which temperature is varied. In the following section we discuss the equations that are valuable in describing the nonlinear, nonexponential structural recovery of glass forming systems.

## 3. The Physics of Structural Recovery

Prior to being able to describe the viscoelastic response of polymeric glasses, it is essential that the underlying physics of the polymer glass be understood. In this section we present the phenomenological equations that have been developed and are generally agreed upon as describing reasonably well the structural recovery of glasses. It is anticipated that the physics incorporated into these equations are the minimum needed to successfully describe glassy behavior, at least to a first approximation. The ideas presented here will carry over to the viscoelastic response of polymers and the phenomenon of physical aging. These will be visited in a subsequent section.

### 3.1 Requirements of the Models

From the above discussion it is readily seen that the kinetics of structural (volume, enthalpy) recovery in glassy materials exhibits a richness of behaviors that offers a severe test of any model. In 1971 Narayanaswamy [9] developed a formalism analogous to that of the reduced variables approach used in viscoelasticity theories to describe “time-temperature” superposition. Importantly, his model incorporated the concepts described above of a distribution of relaxation times (or nonexponential relaxation) and the dependence of the internal material “clock” on the current structure of the glass. Until then there was no model that incorporated the “essential ingredients” described above that are necessary to explain the major features of the phenomenology of glassy kinetics. In subsequent work Moynihan, et al. [10] enlarged upon the Narayanaswamy approach and Kovacs, et al. [11] arrived at similar results independently. The Narayanaswamy [9]-Moynihan [10] model is given an excellent review by Scherer [5] and the Kovacs, Aklonis, Hutchinson, Ramos (KAHR) [11] model is summarized by McKenna [3]. Here we treat them briefly.

### 3.2 The KAHR Model

Following the KAHR [11] approach, we use a multiple ordering parameter description of the (volume) departure from equilibrium. Then the rate of recovery at constant pressure is

$$-d\delta_{i}/dt=-q\Delta \alpha_{i}+\delta_{i}/\tau_{i}\left(1⩽i⩽n\right)$$ (1)

where *δ* is the total departure from equilibrium. Δ*α* = (*α*_l_ − *α*_g_) is the change in coefficient of thermal expansion from liquid (l) to glass (g) at the glass transition and the Δ*α_i_* =*g_i_*Δ*α* are the weighted contributions of each ordering parameter to Δ*α* with

$$\sum_{g_{i}}=1$$ (2)

*q* = d*T*/d*t*, *t* is time and the *τ_i_* are the retardation times associated with each retardation mechanism or ordering parameter. The model assumes that each retardation mechanism is independent of the others and depends on the total departure from equilibrium. Furthermore the total departure from equilibrium is written as

$$\delta =\sum \delta_{i}.$$ (3)

Similar equations can be written for the enthalpy and also for pressure dependence rather than temperature.

The solution to Eq. (1) now depends upon the specific temperature and structure dependences of the *τ_i_*. These dependences are put into the KAHR model in a manner equivalent to the time-temperature superposition principles of viscoelasticity theory. Then, KAHR assume that, by a change in temperature or *δ*, each retardation time is shifted by the same amount and that the amount of shift due to a change in temperature is independent from that due to the departure from equilibrium, i.e., structure. As a result, the spectrum of retardation times simply shifts along the time axis but does not change in shape:

$$\tau_{i}\left(T,\delta \right)=\tau_{i,r}a_{T}a_{\delta }$$ (4)

where *a_T_* and *a_δ_* are the appropriate shift factors for the spectrum at any *T* and *δ* relative to *τ_i_*,_r_ in the reference state at equilibrium. Then simply

$$a_{T}=\tau_{i}\left(T,0\right)/\tau_{i}\left(T_{r},0\right)$$ (5)

$$a_{\delta }=\tau_{i}\left(T,\delta \right)/\tau_{i}\left(T,0\right)$$ (6)

where *T*_r_ is a reference temperature and *δ* = 0 denotes equilibrium. The thermal history dependence of *δ* can be written in a compact form by using a reduced time variable *z*

$$z=\int_{0}^{t}\frac{d\xi }{a_{T}a_{\delta }}.$$ (7)

The relevant expression for *δ*(*t*) is then given by the convolution integral

$$\delta \left(z\right)=-\Delta \alpha \int_{0}^{z}R\left(z-z\right)dz^{\prime }\frac{dT}{dz^{\prime }}$$ (8)

where *R*(*z*) is a normalized retardation (recovery) function for the system

$$R\left(z\right)=\sum_{g_{i}}\exp\left(-z/\tau_{i,r}\right).$$ (9)

In the case of the Narayanaswamy [9]-Moynihan [10] development, the retardation function is expressed in the so-called Kolrausch [7]-Williams-Watts [8] (KWW) function

$$R\left(z\right)=\exp\;{\left(-z/\tau_{r}\right)}^{\beta }.$$ (10)

Importantly, these equations provide a formalism for the description of the behavior of the glass once one determines the functions for *a_T_*, *a_δ_*, Δ*α*, and *R*(*z*). This is not a trivial experimental task and requires some further simplifications. However, the major features of the volume recovery response are reasonably well represented within this formalism. A thorough discussion of the quality of such models is presented by McKenna [3]. At this point we leave the reader to note the importance of Eqs. (1–10) lies in the ability to obtain the nonlinear behavior manifested in the asymmetry of approach experiment simply by including in Eq. (8) the fact that *δ* depends upon itself through the reduced time *z* expressed in Eq. (7). Similar concepts of a material clock that depends upon the response that one is attempting to measure has also become increasingly important in the description of the nonlinear viscoelastic properties of polymers and will be touched upon in a future section.

## 4. Physical Aging in Polymer Glasses

### 4.1 The “Classical” Picture of Aging

#### 4.1.1 The Linear Viscoelastic Regime

It has long been known that polymeric materials are not in equilibrium in the glassy state and that their properties resultingly changed with time. In the early 1960’s Kovacs, Stratton and Ferry [12] attempted to use the ideas of volume recovery and reduced time to describe the behavior of a polymer glass when subjected to changes in temperature. Their results were inconclusive, possibly due to lack of precision in the measurements, but suggested that as a first approximation the idea that as the glassy structure changes, the mechanical viscoelastic response might shift in a fashion similar to the volume recovery response described above. Subsequently, this idea was exploited extensively by Struik [13] in the mid-1970s in his development of the “classical” picture of physical aging. We review this here.

If one subjects the glass to a temperature jump from above *T*_g_ to below *T*_g_, the volume recovery described previously is accompanied by changes in the viscoelastic response of the glass. In Struik’s work, when the mechanical deformations were small, he observed that the creep curves obtained at different aging times *t*_e_ after the T-jump could be superimposed by a shift *a*_te_ along the time axis. He thus established a time-aging time superposition principle similar to time-temperature superposition. Note that we have used the notation *a*_te_ here rather than *a_δ_* as was done above. The reason for this is our feeling that the effects of structure on the structural (volume, enthalpy) recovery response need not be the same as for the viscoelastic (creep, relaxation) response. Thus, although the changing thermodynamic state obviously impacts the kinetics of both structural recovery and creep or stress relaxation, its impact need not be the same for the different processes [14–20].

**Fig. 8:**
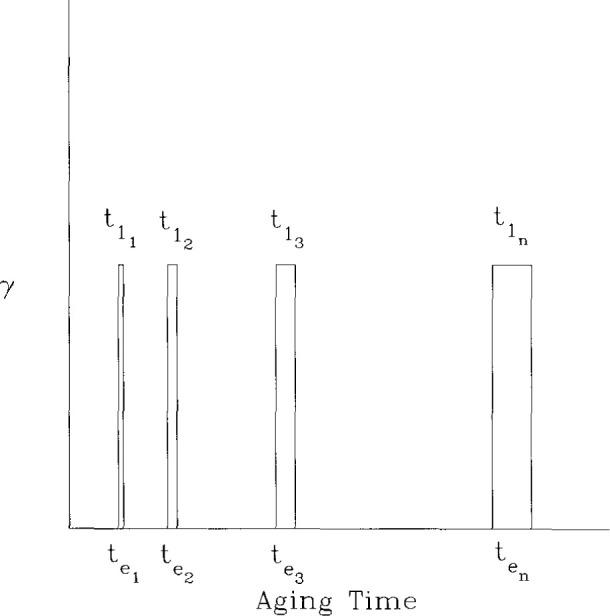
Schematic of loading sequence to probe physical aging after a temperature jump, *γ* is the applied load or deformation. The t_ei_ are the aging times after the temperature change. The *t*_1i_ represent the duration of the load application. In general one selects *t*_1i_/*t*_ei_ ⩽ 0.10.

**Fig. 9:**
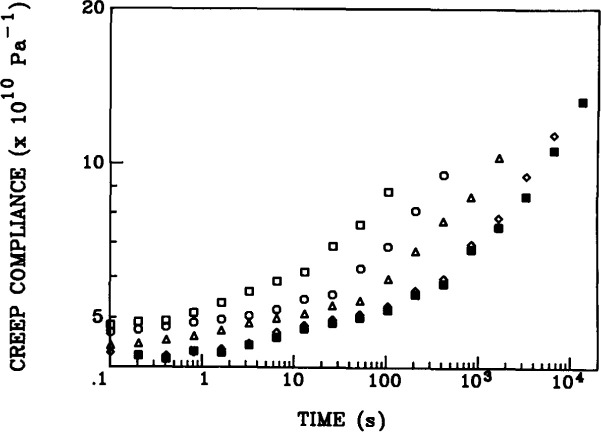
Creep compliance curves at different aging times to for an epoxy glass quenched from *T*_g_ + 22 °C to *T*_g_ − 9 °C. *t*_e_*:* (□) 28 min; (○) 126 min; (Δ) 503 min; (◊) 2013 min; (■) 4026 min. (After Ref. [14b].)

**Fig. 10:**
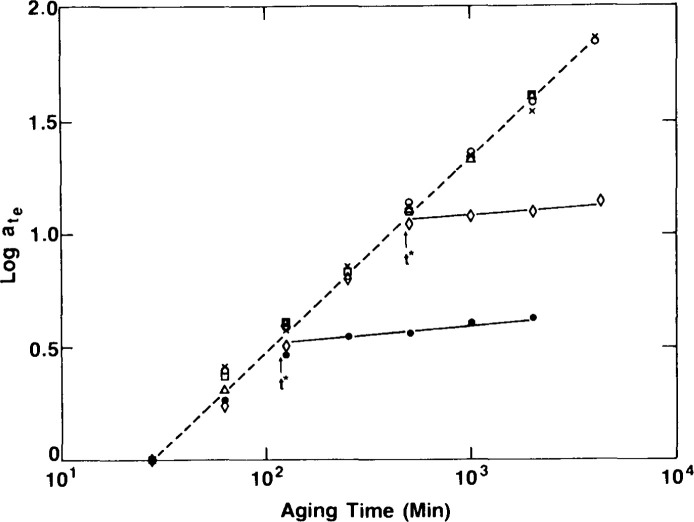
Double logarithmic representation of aging time shift factor *a*_te_ vs aging time *t*_e_ for an epoxy glass aged at different temperatures below its *T_g_*. *T*_g_−*T*: (●) 30.1 °C; (X) 24°C; (□) 20.8 °C; (◊) 10.3 °C; (●) 6.3 °C. (After Ref. [14a].)

Results for aging of epoxy glasses obtained in this laboratory following Struik’s methods are shown in Figs. 8, 9, and 10. Figure 8 first schematisizes the typical experimental procedure in which the polymer is quenched to some temperature at *t*_e_ = 0 and then load-unload “probes” are applied sequentially to the sample at increasing aging times. The duration of the loading time *t*_1_ is kept at less than 0.1 *t*_e_ in order that the changes that occur in polymer structure during the mechanical experiment be small and not influence the measurement. In Fig. 9 we show creep curves obtained from uniaxial extension measurements on an epoxy glass at different aging times in which the shifting of the responses to longer times with increasing *t*_e_ is clear. Typical shift factors required to superimpose such curves plotted against aging time are depicted in Fig. 10 for different temperatures of aging. The important feature that is shown here is that upon aging below the glass transition two types of behavior are evident. First, far below *T*_g_ the aging continues for the duration of the experiments. Second, near to *T*_g_ the aging seems to nearly cease at some time “*t*^†^” that might be expected to be some equilibration time for the glass. This will be discussed subsequently.

In the linear viscoelastic regime Struik found that the double logarithmic shift rate *µ* = d log(*a*_te_)/d log(*t*_e_) ⩽ 1 in the aging regime which he defined as being between the *α* and β transitions in polymers. While this may be a predominant effect, there is little doubt that aging does occur below the *β* transition in glassy polymers, but the relative effects of the structural recovery on the β-transition and the glass transition are different and certainly the time-aging time superposition will break down in this regime. An example of such a problem arose in the work of McKenna and Kovacs [21] on PMMA in which it was found that the stress relaxation responses at different aging times could not be superimposed below 80 °C and this was attributed to the influence of the strong and broad β relaxation in PMMA.

The picture of physical aging that emerges from the work of Struik and others in the linear viscoelastic regime is relatively straight forward and indicates a “structural” dependence of the characteristic viscoelastic times that can be described by a time-aging time superposition principle — at least to first approximation. Struik [13], Kovacs, Stratton and Ferry [12], among others, explain the structural changes in terms of free volume concepts. Because these are well covered in the literature (see, e.g., Ferry [2]; McKenna [3]) we will not discuss them here.

#### 4.1.2 The Nonlinear Viscoelastic Regime

The picture of physical aging put forth above applies to the linear viscoelastic or small stress and deformation regime. In the case of the effects of structural recovery on the nonlinear viscoelastic response of the glass, the picture changes, and as will be seen is controversial. First we will present the views of Struik [13] as originally put forth in his book and early papers. This will be followed by a section describing work done in our laboratory that verify the experiments of Struik (and others) but that provide evidence that contradicts his interpretations.

**Fig. 11:**
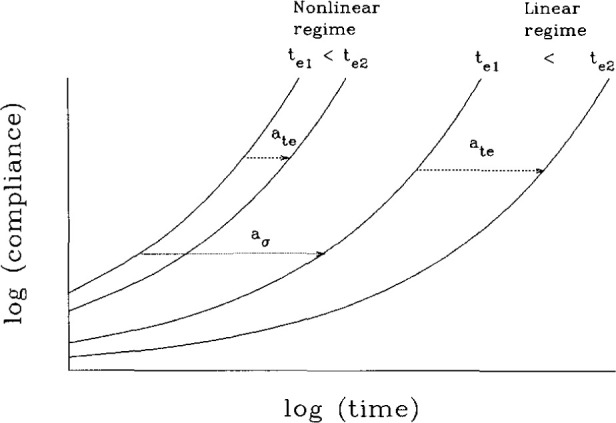
Schematic representation of the relative effects of structural recovery on the small stress (linear regime) and high stress (nonlinear regime) creep compliance of a glassy polymer. The *a*_te_ represent aging time shift factors. The *a*_σ_ is a possible stress shift factor.

**Fig. 12:**
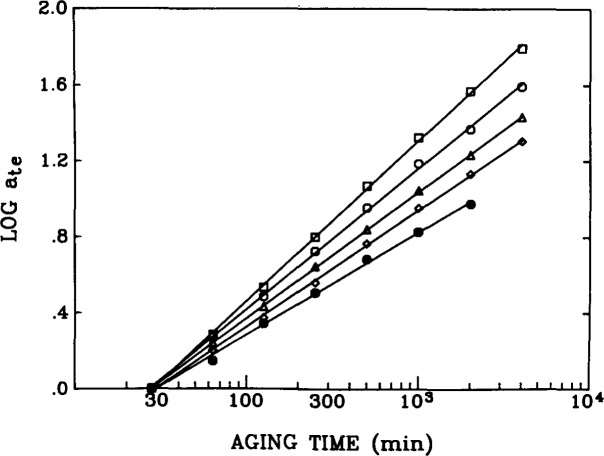
Double logarithmic representation of *a*_te_ vs *t*_e_ for an epoxy glass aged at *T*_g_ −13.2°C. Symbols represent results from tests in which aging response was “probed” at different levels of applied stress: (□) 1 MPa; (○) 5 MPa; (Δ) 10 MPa; (◊) 15 MPa; (●) 20 MPa. (After Ref. [14b].)

**Fig. 13:**
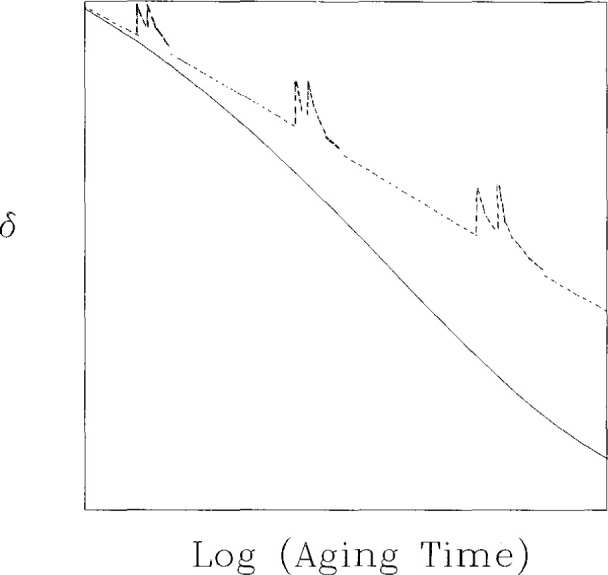
Schematic of anticipated effect of applied stress on the volume recovery response if “rejuvenation” or “erasure” hypotheses are correct. Solid line, volume recovery in no-stress experiment. Dashed line represents residual effect due to erasure or rejuvenation. (See text for discussion.)

Figure 11 depicts schematically the evidence that Struik had that indicates the differences in the aging behavior in the linear and nonlinear viscoelastic regimes. There are two things to be observed here. First, as observed previously by Matsuoka [22], at a given aging time the creep compliance at large stresses is shifted to shorter times relative to the small stress response. Second, upon increasing the aging time, the aging time shift factor is greater for the small stress experiment than for the large stress experiment. Thus, as shown in Fig. 12, when the stress is increased, the slope of the double logarithmic plot of *a*_te_ vs *t*_e_ decreases. Struik concluded from these (and some other experiments) that the large stress erases the prior aging. This has subsequently come to be known as rejuvenation. Figure 13 depicts schematically the sort of interpretation that Struik used to explain why the large stresses or deformations rejuvenate the glass. He hypothesized that the volume (or free volume) is increased by the input of mechanical work by the loading and unloading of the sample with the result that the stresses move the glass closer to the same structural state as the unaged or freshly quenched glass.

These results, and others presented by Struik and others, are not questioned. What is observed as the signature of rejuvenation is the decrease in the slope *μ* of log(*a*_te_) vs log(*t*_e_). However, the hypothesis that the structure of the glass (as the nonequilibrium thermodynamic state) changes upon application of the large stresses or deformations, has specific implications for the aging behavior as manifested in the evolution of the viscoelastic properties as well as for the volumetric response after the material has been deformed. These are addressed in the next section.

### 4.2 Some “Non-Classical” Aging Experiments

In dealing with the aging problem, and in particular with the problem of the interactions of the large deformations or stresses with the volume or structural recovery, we have attempted to address-the problem from the point of view of nonlinear viscoelasticity. In several papers [14–18, 23], we argued that the apparent rejuvenation of the polymer glass was simply a manifestation of the fact that the observed effects were either due to a “memory” effect or, as in the case of the above experiments, of the fact that the volume recovery impacted the large deformation response less than it did the small deformation response. In what follows we show results from two types of experiment that support our contention that aging affects the nonlinear response less than it does the linear response, as evidenced by the Struik observation of a lower value of the double logarithmic derivative *μ* = d log(*a*_te_)/d log(*t*_e_). First, we look at results from experiments performed close to the glass transition and ask what does “*t*^†^” do as a function of stress magnitude? Second, in a similar temperature regime, we show results from experiments using the NIST torsional dilatometer [24], that show no change in the kinetics of volume recovery upon application of large deformations.

**Fig. 14:**
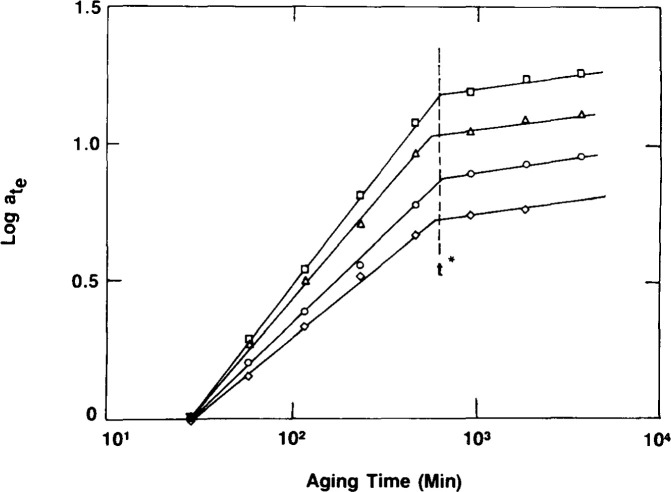
Double logarithmic representation of *a*_te_ vs *t*_e_ for an epoxy glass aged near to the glass transition. Symbols represent tests performed at different applied stresses: (□) 1 MPa; (Δ) 5 MPa; (○) 10 MPa; (◊) 15 MPa. Note that t^*^ (or t^†^) is insensitive to the level of applied stress. (After Ref. 14b.)

Recalling that *t*^†^ is a measure of the aging kinetics (how long until the mechanical response attains its equilibrium response)^1^, the rejuvenation hypothesis suggests that *t*^†^ should increase as the level of the applied “probe” stress *a* increases. With this in mind, Lee and McKenna [14] undertook a series of uniaxial extension creep experiments using epoxy glasses that could be readily tested near to their glass transitions. By changing the magnitude of *σ* in aging experiments after a temperature jump they were able to observe how *t*^†^ varies with *σ* and the change in *μ* at the same time. Figure 14 illustrates their dramatic finding that *t*^†^ is independent of the applied stress *σ* even though the signature of rejuvenation, that *μ*. decreases with increasing *σ*, remains. This result provided strong evidence of the fallacy of the rejuvenation hypothesis, but was indirect in that *t*^†^ is not necessarily the equilibration point for the glass. Therefore, direct and simultaneous measures of the volume recovery and viscoelastic response after temperature jumps were perceived to be necessary. Such experiments are discussed next.

At NIST a torsional dilatometer was built [24] in which the torque, normal force and volume change in samples could be measured simultaneously in arbitrary thermo-mechanical histories. In this work we chose to first examine the down-jump aging experiment in mechanical histories similar to those defined by Struik [13], but in stress relaxation rather than creep. The torsional geometry of deformation was chosen because volume changes due to the mechanical deformation are second order effects in torsion and would therefore not swamp the small volume changes observed after small temperature jumps. (Had extension or compression been chosen, the Poisson’s effect would have been very large compared to the volume recovery observed due to the temperature jump and the problem of taking small differences between large numbers would have magnified any uncertainties in the results). The NIST dilatometer has been described elsewhere [24]. It has a volume change sensitivity of approximately 10^−7^ cm^3^/cm^3^ for short term tests where temperature variations are unimportant. The apparatus now has a long term temperature stability of approximately ± 8 mK which leads to a long term volume stability of about 1.5×10^−5^ cm^3^/cm^3^.

**Fig. 15:**
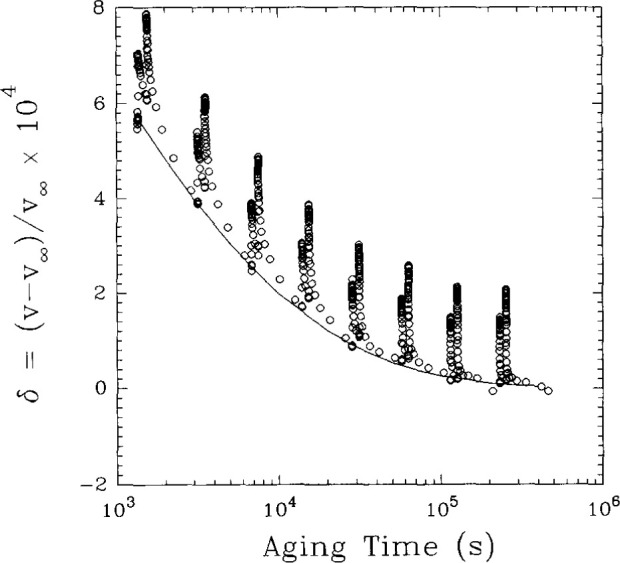
Volume departure from equilibrium vs aging time for an epoxy glass at *T*_g_ −8.9°C after a down-jump from above *T*_g_. Points are data obtained for a torsional “probe” strain of 3%, solid line represents data obtained for an undeformed sample. (Data from Ref. [9].)

Figure 13 shows the sort of dilatometric behavior one would expect during an aging experiment in which the deformations were large. In Fig. 15 typical results from experiments performed in the NIST torsional dilatometer at a large deformation (well into the regime where the “signature” of rejuvenation is seen) are shown. While the application of the torsion does temporarily increase the volume of the glass, as does the reduction of the torsion to zero, what is observed is that the underlying (baseline) volume recovery is unchanged by the application of the large deformations. Thus, the changing aging time shift factor with increasing stress is not explained by a change in the structure of the glass upon application of the deformation. Apparently the volume increases upon torsion do not contribute to a change in the structural recovery rate in the way anticipated by the rejuvenation model. These results indicate that, in some sense, the thermal volume and the mechanical volume are only coupled in the direction that the thermal volume (structure) impacts the mechanical response of the material. On the other hand, the mechanical deformations do not change the underlying structure of the glass in as much as the volume recovery kinetics are unaltered by them. An extensive discussion of this decoupling is presented in Lee and McKenna [14]. Two cautions need to be made about this interpretation of the results. First, volume is not the only state variable which is involved and it is conceivable that the results are only relevant to volume and not, e.g., entropy (enthalpy)—in which case free volume models become very tenuous indeed. Second, the results obtained to date are for behavior below the yield of the polymer and need to be extrapolated with caution beyond the yield.

## 5. Impact of Structural Recovery/Aging on Engineering Properties

Before going on to discuss some of the ways in which the above findings impact the development of methods for the description the mechanical response of glass forming polymers, we take an aside here to describe the impact of aging on some other properties of polymers. In particular, we are interested in the response of engineering properties that often are assumed to be constant. The aging picture presented above provides a basis for understanding qualitatively what is happening. Much remains to be done to explain quantitatively the impact of aging on the yield and failure responses of polymers.

### 5.1 Yield

**Fig. 16:**
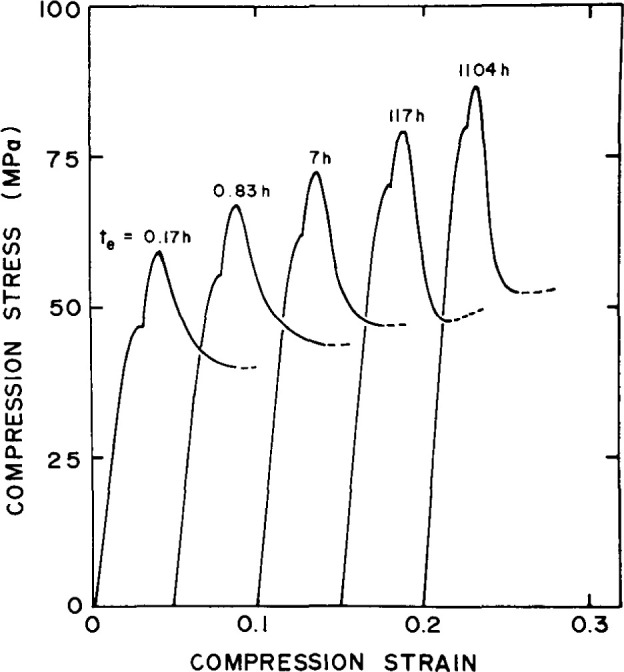
Typical compression curves obtained at *T*_g_ −10 °C for an epoxy glass for different aging times, as indicated. (After Ref. [27].)

**Fig. 17:**
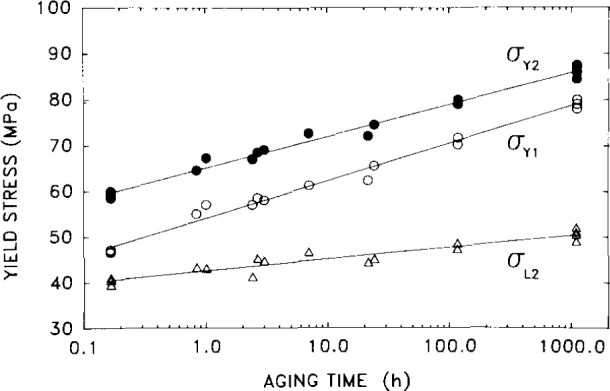
Influence of aging time on the compression yield stress obtained at different strain rates for an epoxy glass tested at *T*_g_ −15 °C. σ_Y1_ represents yield at a strain rate of 10^−3^ s^−1^; σ_Y2_ represents a strain rate of 10^−2^ s^−1^. σ_L2_ is the lower yield after σ_Y2_. (After Ref. [27].)

It has been shown by other workers that the yield stress of a glass forming polymer increases with increasing aging time after a quench [13, 25–26]. Here we describe work performed in our laboratory on epoxy resins that illustrates dramatically the impact of aging on material response [27]. Experiments were performed in uniaxial compression using a technique often used in metal plasticity in which the sample was loaded at one strain rate *ϵ*_1_ until a maximum occurred in the stress strain curve. At the stress maximum the strain rate was increased by a factor of 10 to *ϵ*_2_. The procedure allows one to obtain data at two strain rates using a single specimen. Figure 16 depicts the responses of an epoxy glass at Increasing aging times after a quench from above *T*_g_ to a temperature near to the glass transition. It is obvious from this plot that the yield stress at both strain rates increases as the aging time increases. The magnitude of the increase is surprising, and as seen in Fig. 17 can attain a factor as great as 1.8 in aging from 0.1 h to 1000 h. Such behavior was also observed for a thermoplastic by Pan and McKenna [28] in separate experiments. Interestingly, in other experiments nearer to *T*_g_ one also observes *a t*^†^ break in the evolution of the yield stress with aging time. It turns out that this break occurs at longer times than for the viscoelastic properties. [16, 27]. This question of different time scales for different properties is one which is perplexing and unresolved. (A discussion of different time scales in polymer glasses is made in the paper of Santore, Duran and McKenna [16]). The major observation here is that the yield stress is obviously dramatically affected by the structural recovery of the glass and can evolve for long periods of time. This obviously can be important for engineering structures and composites.

### 5.2 Creep Rupture

The failure of materials is another time dependent property of polymers that has only partly been explained in spite of much work in the area. In the case of the impact of structural recovery on failure, there has been little work done on failure of bulk materials in creep rupture conditions. In studies performed by Crissman and McKenna [29, 30] on poly(methyl methacrylate) (PMMA) it was found that under certain conditions the creep rupture life-time could be related to the viscoelastic properties of the material. In this case the impact of aging on both the creep response and the failure response was similar.

Two studies were carried out. First, PMMA that had been aged at room temperature for approximately 5 years was compared with PMMA aged for one week at the same temperature. Second, PMMA samples that had been aged at 80 °C and then tested at 22.5 °C were compared for different aging times with the result that chemical and physical aging responses were both present.

**Fig. 18:**
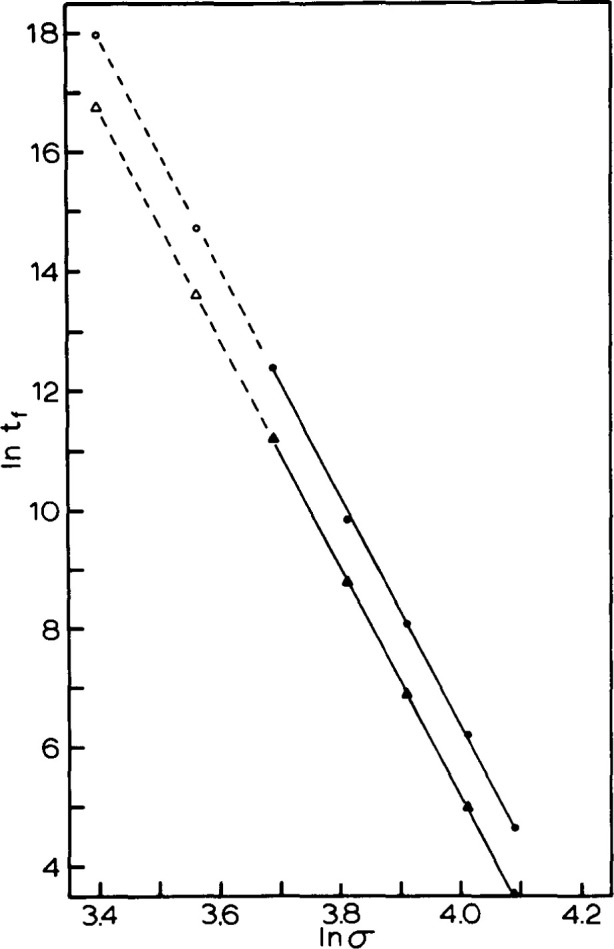
Double logarithmic representation of the creep rupture time *t*_f_ vs applied stress for poly(methyl methaciylate) tested at 22.5 °C. Triangles represent data obtained for freshly quenched samples. Circles are for samples aged at room temperature for 5 years. (After Ref. [29].)

**Fig. 19:**
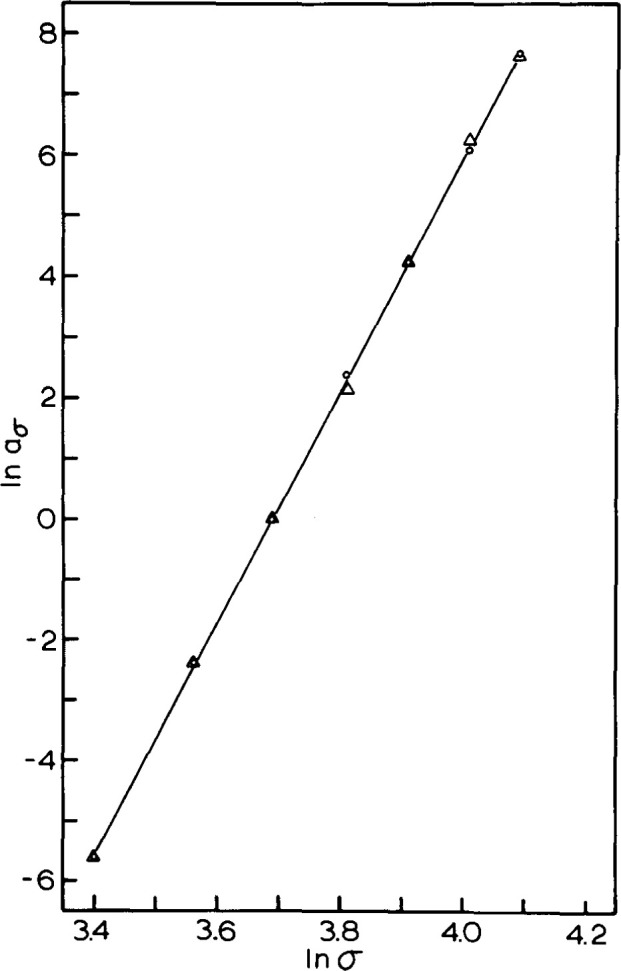
Double logarithmic representation of the stress shift factor vs applied stress for poly(methyl methacrylate) tested at 22.5 °C. Symbols as in Fig. 18. (After Ref. [29].)

In Fig. 18 we depict the times-to-failure *t*_f_ vs *σ* for the 5 year aged sample and for the 1 week aged sample. Two things should be noted from this figure. First, the stress dependence is a power law with an exponent of approximately 20. Second, the failure time for the 5 year aged sample is approximately 2.5–3 times that of the freshly quenched sample. In Fig. 19 we depict the time-stress shift factors *a_σ_* vs *σ* for the same two polymers obtained from creep curves obtained at different stresses. Again the response is power law in stress and the power law exponent is again of magnitude 20. The similarity in the exponents is evidence that the viscoelastic and failure responses are related. In addition, we found that the aging time shift between the aged and unaged glasses was a factor of 2.5–3.

When time-aging time superposition also holds, as it did here, the failure and viscoelastic properties are readily described by the same shift factors. Thus, the failure and viscoelasticity of the PMMA are apparently impacted in the same way by the structural recovery.

In the instance of the PMMA we found that the strain to failure was virtually unchanged as a function of stress or whether the sample had been aged or not. This led us to define some conditions in which a common failure criterion would be valid. The failure criterion is that the strain rate at failure multiplied by the time to failure are constant:

$${\dot{\epsilon }}^{\prime }t_{f}=c.$$ (11)

This was found to be true under the following conditions:

1. The time-stress superposition holds in a way such that the horizontal shift of the creep compliance curves is accompanied by a vertical shift *b_σ_* = *σ/σ*_r_ where *σ*_r_ is the reference stress.
2. The strain at failure is a constant.

**Fig. 20:**
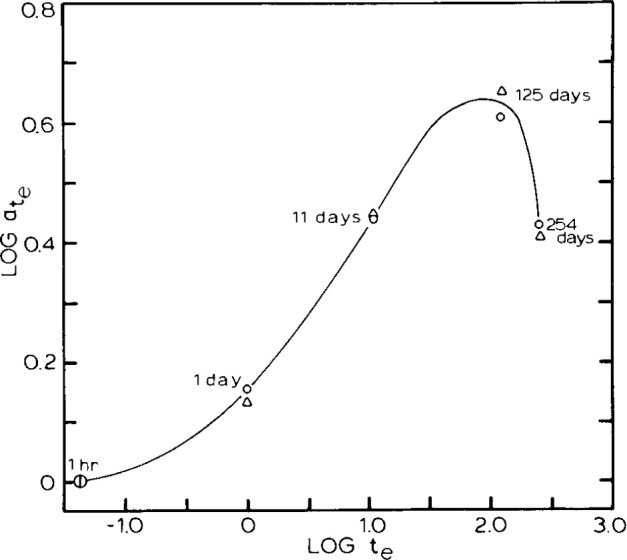
Double logarithmic representation of the aging time shift factor $a_{t_{e}}$ vs aging time *t*_e_ for a poly(methyl methacrylate) glass aged at 80 °C and tested at 22.5 °C. Peak in data correlates with onset of chemical degradation (see text for discussion; after Rcf. [30]).

This work was extended [30] by examining the aging of the same PMMA at 80 °C. There were three important results that came from this work. First, for a given aging time at 80 °C, the same type of time-stress superposition at 22.5 °C was observed as above. Also, the 22.5 °C creep responses of all of the samples aged at 80 °C could be superimposed by time-aging time superposition. However, the samples aged at 80 °C and tested at 22.5 °C did not show superposition with the samples aged and tested at 22.5 °C. This result is not fully understood. The second point is that at each aging time the failure criterion of Eq. (11) was followed. However, *c* was a function of aging time. Therefore, the aging time shift factors did not describe the changes in failure times, as they had in the samples aged at 22.5 °C. Finally, as shown in Fig. 20 the chemical degradation of the samples began to have an impact not only on the failure strain, but also on the shift factors. Thus, $\log\left(a_{t_{e}}\right)$ vs log(*t*_e_) goes through a maximum that corresponded reasonably well with the changes in molecular weight, which was felt to possibly result in some plasticization of the material. The maximum also corresponded to changing density for samples that had been aged for a long time. Molecular weight and density changes for the samples are shown in Table 1.

It is obvious that the complete understanding of the impact of structural recovery on the failure of polymers is not currently at hand. Recent work by Arnold [31] has shown that the increasing lifetime observed on aging of the PMMA (at 22.5 °C) is not repeated for polystyrene. This may not be surprising given the strong correlation between the viscoelastic and failure responses of PMMA, which have yet to be demonstrated for other polymers. However, this is an important effect and the physics governing it are not nearly as well defined as are those that describe the structural recovery itself. Finally, to our knowledge there has been little work performed to examine the impact of aging on the fatigue lifetimes of polymers under dynamic loading conditions.

### 5.3 Implications for Performance of Composite Materials

The performance of composite materials is the result of a complex interaction among the reinforcing fiber, the polymer matrix and the fiber/matrix interface [32]. In fact, in many senses composites are not materials but rather material systems or structures. Then good design must include a thorough understanding of the behaviors of the individual components as well as the ways in which they interact. Thus, for example, the interlaminar properties of a composite may be resin or matrix dominated, but restraints imposed by the stiff fibers will influence the matrix and interface properties. Because the composite structure is complex and behavior depends upon the specifics of each system, in the following paragraphs we will simply make a general case that structural recovery or aging of the resin can be expected to significantly impact the performance of many, if not all, composite systems.

**Fig. 21:**
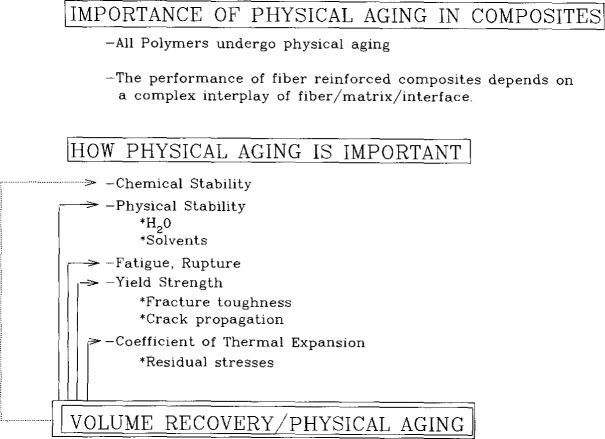
Depiction of importance of structural recovery or physical aging upon the performance of composite materials.

In Fig. 21 we depict the possible interactions between the matrix properties and the potential influence of the aging process on composite behavior. As can be seen, with the exception of chemical aging, which to a first approximation may not be significantly affected by the physical aging process, there are many possible routes for the matrix properties to change with a resulting change in the performance of the composite. Of course, the changes will not always be detrimental. During the course of structural recovery the creep compliance shifts to longer times, which can be expected to improve interlaminar creep resistance and off-axis creep resistance. Similarly, an increase in matrix yield stress may enhance transverse tensile strength. At the same time, interlaminar fracture toughness might be expected to be adversely affected. Finally, as noted in the figure, the fact that the dimensions of the matrix vary with the entire thermal history, can lead to residual stress build-up in composite systems. Understanding how processing histories affect the residual stresses can improve the manufacturing procedure to improve performance. Furthermore, changing the residual stresses and their relaxation behavior can lead to better control of system dimensions over the lifetime of the composite. Below we outline an example in glass to metal seals that illustrates how the understanding of the structural recovery of glasses was used to understand a failure in a hermetic seal.

**Fig. 22:**
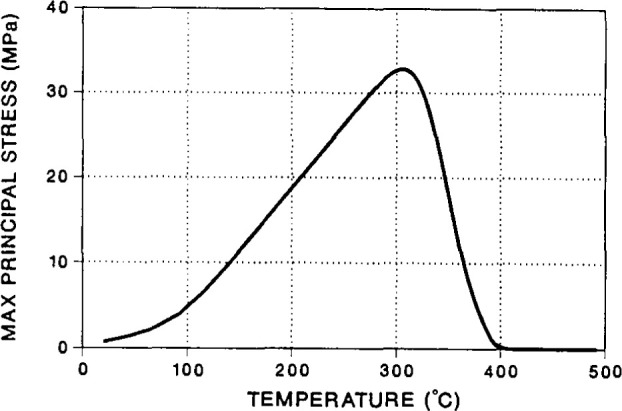
Finite element model predictions of stress history upon cooling of a glass-metal seal. Tensile stress arises due to structural recovery nonlinearities. Thermo-elastic analysis resulted in a compressive stress of −6.7 MPa. (After Ref. [33], with permission.)

In glass to metal seals used in electronics applications for the Department of Energy it is desirable to have the glass that seals the inner metallic core be under a residual compressive stress to prevent long term cracking of the seal. In the case reported by Chambers [33], tensile cracking was occurring in the seal in spite of the thermoelastic analysis prediction that the glass should be under a residual compression. At that time Chambers implemented a finite element analysis that took into account the structural recovery of the glass seal during the cooling stage of the seal formation. For the analysis, the structural recovery was modeled using the Narayanaswamy [9]-Moynihan [10] formulation of the structural recovery physics described above. The nonlinear material response that results upon cooling through the glass transition range was found to cause a large residual tensile stress in the glass at the temperature at which cracking was observed to occur. This is depicted in Fig. 22. The important point here is that a lack of understanding of the phenomenology of the structural recovery that tells that the volume changes are more complex than those given by a simple thermo-elastic analysis (i.e., simply taking the differences in coefficient of thermal expansion) lead to a failed part. Similar effects can be expected for fiber reinforced composites. In the case of the Chambers [33] analysis it was possible to model different processing conditions (cooling histories) that would lead to a significant reduction of the tensile stress and a reduction of seal failure.

## 6. Glassy Physics and the Modeling of the Behavior of Polymers and Composites

From the above discussions it is readily apparent that there is a significant body of knowledge that is available to model the structural recovery of polymeric glasses. Such knowledge is important in the ability to develop computational models that are useful in predicting polymer material and composite system behaviors. In this section we will briefly discuss the development of such models and the ingredients necessary to implement them. Our perspective will begin with the constitutive models that describe the mechanical response of the material. (The reader is reminded that the Narayanaswamy [9]-Moynihan [10] and KAHR [11] models provide the constitutive equations to describe the structural recovery). From there we will discuss some physical evidence that exists to suggest that certain classes of constitutive equations are not correct. We will suggest some areas of work that should be vigorously pursued and finally we will argue that, although the computer power currently available is insufficient to handle the full modeling problem, it soon will be and the technological community needs to be ready to implement current and future knowledge relevant to the models we describe when computing power catches up with our knowledge.

### 6.1 A Brief Look at Some Nonlinear Constitutive Equations

Although there is a considerable body of knowledge concerning the nonlinear response of polymer melts and solutions, much of the work dealing with the behavior of engineering polymers has been devoted to plasticity and yield. Below the yield point these materials are, from this writer’s view, viscoelastic and the correct constitutive description of their behavior will need to fall into that category. Therefore, we will not consider plasticity equations and will limit ourselves to the nonlinear viscoelastic constitutive equations.

The development of nonlinear constitutive equations is a very sophisticated field that we do not intend to thoroughly survey. Furthermore, the general multiple integral equations, while they have been used with success in some cases [34, 35] are very cumbersome to use both experimentally in the number of experiments required for the determination of material properties and computationally. Rather, we want to look at a class of single integral nonlinear constitutive laws that we refer to as reduced time or clock type equations and comment on their potential and the need to further evaluate those that appear to be promising.

The type of equation that describes the nonlinear structural recovery that was discussed previously falls into the category of reduced time equations. The material clock in the Narayanaswamy [9]-Moynihan [10] and KAHR [11] equations depends upon the instantaneous structure of the glass. (See Eqs. [7–10].) Similar classes of equations have been developed for polymer viscoelasticity and we describe those here.

### 6.2 The Schapery Model

One of the first nonlinear viscoelastic models of polymer solids to use reduced time variables was developed by Schapery [36, 37] in the 1960’s and this model is still valuable today because the material response functions can be reasonably easily evaluated in the laboratory and there is reason to believe that computationally it will be relatively easy to implement in finite element codes. Furthermore, the Schapery model has a formulation for both strain as a function of stress history and stress as a function of strain history—a problem that is often difficult to treat in some of the constitutive equations that have been developed. Thus, both creep and stress relaxation can be handled, albeit with different material property functions.

The creep formulation (strain as a function of stress) is as follows:

$$\epsilon =g_{0}D_{0}\sigma +g_{1}\int_{0}^{t}d\tau \Delta D\left(\Psi -\Psi^{\prime }\right)\frac{dg_{2}\sigma }{dt},$$ (12)

where *ϵ* is the strain, *σ* is the stress, *D*_0_ is the zero time compliance, Δ*D* is the time dependent part of the compliance, *τ* is the dummy time variable, the *g_i_*,’s are material parameters. The reduced time arguments are defined by

$$\Psi =\int_{0}^{t}dt^{\prime }/a_{\sigma }\;\left(a_{\sigma }>0\right)$$ (13)

$$\Psi^{\prime }=\Psi \left(\tau \right)=\int_{0}^{\tau }dt^{\prime }/a_{\sigma },$$ (14)

where *a_σ_* is the stress shift factor. A similar set of equations was developed for the relaxation formulation (stress as a function of strain) as

$$\sigma =h_{e}E_{e}\epsilon +h_{1}\int_{0}^{t}d\tau \Delta E\left(\xi -\xi^{\prime }\right)\frac{dh_{2}\epsilon }{d\tau },$$ (15)

where the *h_i_*’s are material parameters and the reduced time arguments are defined as

$$\xi =\int_{0}^{t}dt^{\prime }/a_{\epsilon }\;\left(a_{\epsilon }>0\right),$$ (16)

$$\xi^{\prime }=\xi \left(\tau \right)=\int_{0}^{\tau }dt^{\prime }/a_{\epsilon },$$ (17)

*a_ϵ_* is the strain shift factor.

In the Schapery formulations the reduced times are introduced in a way that is similar to that for time-temperature superposition in the sense that the strain shift factor and the strain or the stress shift factor and the stress both appear on the same side of the equation. Thus, unlike the structural recovery equations in which *δ* depends on temperature history and itself through a*_δ_* [Eqs. (7) and (8)], the nonlinearity is simpler and the equations easier to handle. Also, material nonlinearity is introduced through the material parameters *h_i_* or *g_i_*. These equations have been used with some success and should be further evaluated because of their potential computational ease. There have also been some recent results [38] that indicate the possibility that the Schapery equations may be inadequate without modifications to treat all deformation or stress histories.

### 6.3 The Zapas Strain-Clock Model

One of the most successful nonlinear viscoelastic models used in melt and solution rheology was the BKZ theory of Bernstein, Kearsley and Zapas [39]. In the mid-1970s Zapas [40] proposed a modification of this BKZ model that would include a strain clock reduced time and attempted to apply the equation to the description of solid polymers. The formulation for the modified BKZ is tensorial in nature, for simplicity here we will deal with that in simple shear:

$$\sigma_{12}\left(t\right)=\int_{-\infty }^{t}\left\{[\gamma \left(t\right)-\gamma \left(\tau )\right]G*\left[\gamma \left(t\right)-\gamma \left(\tau \right),\Phi \left(t,\tau \right)\right]\right\}\dot{\Phi }\left(t,\tau \right),\gamma \left(\tau \right),t-\tau )]d\tau ,$$ (18)

where *σ*_12_ is the shear stress, *γ* is the shearing strain, *t* is current time and *τ* is past time. G*(*γ,t*) is the derivative of *G* at the second argument, where *G*(*γ,t*) is the nonlinear relaxation modulus. The strain clock which gives the reduced time is defined as

$$\Phi \left[\gamma \left(t\right),\gamma \left(\tau \right),t-\tau )\right]=\int_{\tau }^{t}\dot{\Phi }\left[\gamma \left(t\right),\gamma \left(\Omega \right),t-\Omega )\right]d\Omega .$$ (19)

The strain-clock in the modified BKZ theory is a function of the entire strain history, as defined by Eq. (19). Note that again the strain-clock function (shift function) appears on the same side of the equation as does the strain — again a different form of nonlinearity from that seen in the Narayanaswamy [9]-Moynihan [10] and KAHR [11] equations for structural recovery. Additionally, the modified BKZ theory shown here does not have a ready creep formulation, which can limit its versatility. As formulated it is also limited to incompressible materials. Both of these problems can be overcome by further development if the model is demonstrated to be useful in laboratory tests. The model will be more difficult to implement than was the Schapery [36, 37] model. It may, however, prove to be more general, although there has been little work done to demonstrate its utility. McKenna and Zapas [41] showed that it could be used to describe the two step behavior of a glassy PMMA in torsion and found that the clock function required to describe the shearing response also described the normal stress response in the same experiment.

### 6.4 The Bernstein-Shokooh Stress-Clock Model

In the Bernstein-Shokooh [42] stress-clock model, again the BKZ theory serves as the starting point. Now, however, the material time is assumed to depend upon the stress. The equations in simple shear can be written as

$$\sigma_{12}(t)=\int_{-\infty }^{t}\left\{G*\left[\gamma (t)-\gamma (\tau ),\beta (t,\tau )\right]\right\}\;b_{\sigma }(\tau )d_{\tau }.$$ (20)

Where now *b_σ_* is the stress shift factor and the reduced time *β*(*t,τ*) is defined as

$$\beta \left(t,\tau \right)=\int_{\tau }^{t}b_{\sigma }\left(s\right)ds.$$ (21)

As in the BKZ theory [39] itself, the Bernstein-Shokooh [42] stress-clock model is formulated for stress relaxation. However, unlike either the Schapery [36, 37] model or the Zapas [40] strain-clock model, the shift factor depends upon the dependent variable as is the case for the Narayanaswamy [9]-Moynihan [10] and KAHR [11] models. Thus, in Eq. (20) we find that the stress depends upon itself through the reduced time. Bernstein and Shokooh [42] showed that if one linearizes the equations in the strain (take the small strain limits), then one can invert Eq. (20) to arrive at a creep formulation as well as the relaxation formulation. Nonlinearities still arise because of the dependence of the time on the stress. The creep equation for simple shear is

$$\gamma_{12}\left(t\right)=1/2\sigma_{12}\left(t\right)J_{0}+1/2\int_{0}^{t}J\left[\beta \left(t,\tau \right)\right]\sigma_{12}\left(t\right)b_{\sigma }\left(\tau \right)d\tau )$$ (22)

and the symbols have the same meaning as above. Note that this is the “linearized” form for the creep, therefore the compliance *J* depends not on strain but on the reduced time only.

The stress-clock formulation gives qualitative behavior that one observes in solid polymers. However, to this author’s knowledge the constitutive law has not been tested for data from creep or relaxation in solid polymers. Laboratory evaluation of the model is obviously required.

### 6.5 Volume Clock Models

There have been several attempts in the literature to use volume (or free volume) clocks to describe the nonlinear viscoelastic behavior of polymers [43–45]. In this paragraph we present the constitutive model developed by Knauss and Emri [45].

Knauss and Emri [45] define a reduced time in terms of a shift factor *ϕ*[*T*, *C*, *σ*] where the functional forms of temperature T, concentration *C* and stress *σ* dependences are formulated from “free” volume considerations. Then one can write the isothermal, nondiluted response as:

$$S_{ij}=2\int_{-\infty }^{t}\mu \left[Z\left(t\right)-Z^{\prime }\left(\xi \right)\right]\frac{\partial e_{ij}\left(\xi \right)}{\partial \xi }d\xi$$ (23)

$$\tau_{KK}=3\int_{-\infty }^{t}K\left[Z\left(t\right)-Z^{\prime }\left(\xi \right)\right]\frac{\partial \theta \left(\xi \right)}{\partial \xi }d\xi ,$$ (24)

where Z is the reduced time, *S_ij_* are the deviatoric stresses, *e_ij_* are the deviatoric strains, *τ_KK_* is the first stress invariant and *μ*(*t*) and *K*(*t*) are material functions. *θ*(*t*) is the dilatational strain in the sample and is related to *τ_KK_* through the bulk creep compliance *M*(*t*) and determines the reduced time *Z*:

$$Z\left(t\right)-Z^{\prime }\left(\xi \right)=\int_{\xi }^{t}\frac{ds}{\phi \left[\theta \left(s\right)\right]}$$ (25)

$$\theta \left(t\right)=1/3M\left(t\right)*d\tau_{KK},$$ (26)

where the * denotes a Stieltjes Convolution operation.

Again we see that material nonlinear behavior can be modeled by the use of a material time that runs differently from the laboratory time. In this case, the volume clock depends upon the hydrostatic pressure [Eq. (26)] which affects both the deviatoric (distortional) components of the stress [Eq. (23)] as well as the dilatational components [(Eq. 24)]. Upon applying Eqs. (23–26) Knauss and Emri [45] show that yield-like behavior can be achieved in simple extension. However, in isochoric motions, such as shear, or volume decreasing motions, such as compression, these equations would not give a yielding response since the material time would either be the same as the laboratory time (shear) or the material time would shift to longer times upon decreasing the volume (compression).

The equations of Shapery [36, 37], Zapas [40, 41], Bernstein [42] and Knauss and Emri [45] all have some similar structures and can reproduce some of the features of nonlinear behavior of glassy polymers. The question arises, of course, of how one distinguishes among these potentially useful material descriptions. Obviously laboratory tests can be used to test them directly but these can be tedious and one would like to have some physical basis on which to select which equations to evaluate. We cannot make this evaluation today. However, in the next section we discuss some of the physics of clocks with the intent of providing some ideas and thoughts that should serve to guide such evaluations.

### 6.6 The Physics of Clocks

In a sense the physics of material clocks is not well developed, although obviously the mathematics is. Schapery’s [36,37] model is developed from considerations of nonequilibrium thermodynamics and the clock functions or shift factors can be related to second and third order terms in the entropy production and the Gibbs free energy. Obviously, further developments in the relaxation of complex systems need to be made before the full physics is understood.

From a phenomenological point of view, the clock idea is straightforward. The molecular mobility that determines the structural recovery, stress relaxation, or creep (i.e., of the system dynamics) is dependent on a state variable, applied stress or strain, strain history, etc., in a specific way. In particular, the relaxation or retardation function that describes the material behavior shifts rigidly along the time axis. If such constitutive laws are to be reasonable descriptions of material behavior, the correct variable that shifts the mobility or time scale of the material must be chosen and the constitutive law must incorporate the shift in the correct way. Because of the complicated nature of the nonlinear phenomena, attempting to validate the clock functions is difficult and there have been few systematic attempts to determine how well these functions work. In fact, here is not the place to make that evaluation. However, we do want to present some data that suggest that the volume clock, while a decidedly attractive approach to nonlinear viscoelastic constitutive law development, incorporates the wrong physics. This is not to say that the others are more correct, it is just that it is a simpler task to test the physics of the volume clock than the others where the physics is more obscure and less readily tested.

### 6.7 Some Evidence Against Volume Clocks

**Fig. 23:**
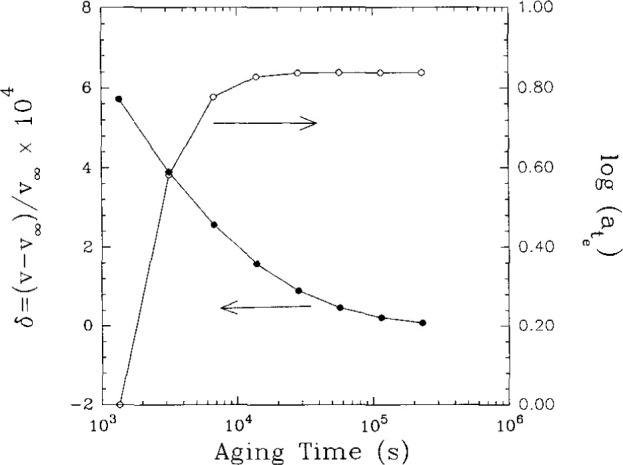
Comparison of aging time shift factor (log *a*_te_) evolution towards equilibrium with that of volume departure (*δ*). See text for discussion. (Data from Ref. [16].)

In fact the evidence against the volume clock was presented earlier in this paper. Recalling the measurements in which we observed the physical aging of an epoxy glass near to the glass transition at the same time that we measured the volume recovery, one can replot the data as log(*a*_te_) vs *δ*. This is done in Fig. 23. As seen in the plot, log(*a*_te_) reaches a (nearly) constant value before the volume reaches equilibrium at *δ*=0. If the volume clock approach were correct, then equilibrium would be attained simultaneously. Obviously there is a lack of one-to-one correspondence between the volume and the evolving mechanical properties. Our suggestion has been that a strain or stress clock might be a better description of the response. These would also explain why such phenomena as the apparent rejuvenation observed by Struik [13] occur in tension, compression and shear where the volume changes are very different. In addition, the stress and strain clocks allow for the description of large deformation behavior such as yield in compression and shear. Quantitative descriptions remain to be tested.

## 7. The Importance of Understanding the Physics of Glassy Polymer

In the above we have reviewed the phenomenology of the structural recovery of polymer glasses (and inorganic glasses as well). The equations that describe the nonlinearities incorporate two major premises. First, the concept of a material clock in which the mobility of the glass (hence the structural recovery kinetics) depends explicity on the thermodynamic state through a shift factor *a_δ_*. Second, there exists a material retardation function that is nonexponential in nature. It has historically been represented as a distribution of retardation times or a stretched exponential KWW function. We have seen how the understanding of how the material properties, through a reduced time, depend upon themselves leads to a description of otherwise complicated nonlinear behaviors.

The nonequilibrium nature of glassy materials impacts significantly the performance of polymer glass formers. Not only does the volume evolution result in a material whose dimensional stability is very problematical, it also leads invariably to changes in the engineering properties of the material. These changes can result in improved or deteriorated performance. Also, we have shown how the inherent instability of glass forming systems can lead to unexpected consequences in composite systems. Unexpected residual stresses, dimensional changes, and the like can all result from the nonequilibrium nature of glassy matrices in composites and other two component systems. Understanding the underlying phenomenology of the glass formers can lead to better process control to minimize problems.

Finally, we discussed the use of material clocks to describe the nonlinear viscoelastic behavior of solid polymers. The equations incorporate material time functions that depend upon stress, strain or volume. They have been shown to mimic, at least qualitatively, many of the observed nonlinear behaviors of polymeric materials in mechanical tests. However, there has been little in the way of systematic evaluation of either the physics behind the clock functions or the range of usefulness of the constitutive equations that result. In the last paragraphs of this paper we argue that the time is right to make those evaluations.

In the example above that showed that the nonlinear behavior of glass forming systems can lead to surprising tensile stresses in glass-metal seals, we presented a vision of the future. In this instance. Chambers [33] and his co-workers were able to use known physics and apply them to a specific problem in a finite element code. Going the next step to more complicated geometries and incorporating the material clock functions into the mechanical behaviors in addition to the volume recovery behavior of the glassy material will require a major effort. If that effort is successful, however, a large benefit will be reaped. In almost all manufacturing of polymers into two component systems, be they composites or electronic packaging, the polymer starts off in a “liquid” state and passes through a glass transition—either thermally or chemically. The parts then are cycled thermally and internal stresses (residual stresses) build up in the systems. These stresses can lead to failure (as in the glass-metal seal example) or to problems with dimensional stability or both. Successful implementation of computer codes to describe this behavior is important and possible. It will provide the ability to design processes in a way that reduces the number of iterations required to manufacture a component with “zero” defects. The barriers to the implementation of codes for complex parts are the following: 1) Computer power; 2) Acceptance and validation of appropriate nonlinear constitutive laws; 3) Lack of physical data (measurements) for the materials.

As long as the computer power has been insufficient to implement codes with sufficient complexity to reflect the true nature of the problem, we have been reluctant to move forward in the full development of the appropriate constitutive models. Now the computer power is catching up. The glass-metal seal problem is a simple one, but incorporates part of what is required—the nonlinear phenomenology of the Narayanaswamy [9]-Moynihan [10]-KAHR [11] models of structural recovery. The nonlinear stress and strain viscoelastic constitutive laws could be incorporated into codes for simple systems as well. However, the constitutive laws need to be validated. This means testing the categories described above in the range of temperature, stress and strain relevant to processing of polymers and their composite systems. From the point of view of computing these cannot be fully implemented for complex systems at this point in time. However, it would be a shame to begin the evaluation of such constitutive laws only after ten years or so when computers will be able to do the calculations. Therefore, we argue that there is a need for a coordinated effort to 1) examine the physics of material clocks; 2) establish experimentally their ability to describe the nonisothermal, nonlinear creep and relaxation of glass forming polymers (both amorphous and semicrystalline) in multiple deformation geometries (tension, compression and shear); 3) develop efficient computer codes that can be vahdated in simple two component systems. Then, when computer power reaches its next levels, it will be a relatively straight forward task to implement finite element codes for complex problems. Current knowledge is now sufficient to make this breakthrough. The only thing that is lacking is the will and coordination to do it.

## Figures and Tables

**Table 1:** Apparent molecular weights and densities of PMMA samples after thermal treatment

Thermal treatment	*M*_n_ b	*M*_w_ b	Density (g/cm^3^)^c^
As received	4.85×10^5^	8.22×10^5^	1.1887
Quenched from 120 °C to 23 °C	4.42×10^5^	8.13×10^5^	1.1887
Aged 1 h at 80 °C	4.52×10^5^	8.18×10^5^	1.1884
Aged 11 d at 80 °C	4.26×10^5^	7.8×10^5^	1.1889
Aged 125 d at 80 °C	3.02×10^5^	5.62×10^5^	1.1905
Aged 254 d at 80 °C	2.54×10^5^	5.03×10^5^	1.1888

a The samples were initially heated to 120 °C for 1 h followed by quenching to 80 °C.

b The relative error in the determination of these quantities is estimated to be about 10%.

c Estimated relative error in measurement is 0.0003 g/cm^3^.

## Footnotes

1 In the prior work from this laboratory cited here we referred to *t*^†^ at *t^*^*. Here we have adopted the notation *t*^†^ because it was brought to our attention that *t*^*^ is often used to mean reduced time in some other milieu. Hence we seek to avoid future confusion by introducing *t*^†^.
